# The emerging role of microtubules in invasion plasticity

**DOI:** 10.3389/fonc.2023.1118171

**Published:** 2023-02-13

**Authors:** Anna Legátová, Markéta Pelantová, Daniel Rösel, Jan Brábek, Aneta Škarková

**Affiliations:** ^1^ Department of Cell Biology, Charles University, Prague, Czechia; ^2^ Biotechnology and Biomedicine Centre of the Academy of Sciences and Charles University (BIOCEV), Vestec u Prahy, Czechia

**Keywords:** microtubules, invasion plasticity, amoeboid, mesenchymal, cancer, 3D migration

## Abstract

The ability of cells to switch between different invasive modes during metastasis, also known as invasion plasticity, is an important characteristic of tumor cells that makes them able to resist treatment targeted to a particular invasion mode. Due to the rapid changes in cell morphology during the transition between mesenchymal and amoeboid invasion, it is evident that this process requires remodeling of the cytoskeleton. Although the role of the actin cytoskeleton in cell invasion and plasticity is already quite well described, the contribution of microtubules is not yet fully clarified. It is not easy to infer whether destabilization of microtubules leads to higher invasiveness or the opposite since the complex microtubular network acts differently in diverse invasive modes. While mesenchymal migration typically requires microtubules at the leading edge of migrating cells to stabilize protrusions and form adhesive structures, amoeboid invasion is possible even in the absence of long, stable microtubules, albeit there are also cases of amoeboid cells where microtubules contribute to effective migration. Moreover, complex crosstalk of microtubules with other cytoskeletal networks participates in invasion regulation. Altogether, microtubules play an important role in tumor cell plasticity and can be therefore targeted to affect not only cell proliferation but also invasive properties of migrating cells.

## 1 Introduction

Cells have adopted various migration/invasion modes which vary in their nature of force generation and dependency on cell-cell and cell-extracellular matrix (ECM) adhesion. Collective migration requires both cell-cell and cell-ECM adhesion, mesenchymal migration omits intercellular adhesion but strongly relies on cell-ECM contact and amoeboid migration can be independent of adhesion altogether. The large range of invasion modes ensures physiological migration of cells in various environments, but in the hands of cancer cells it has become a dangerous trait. The ability of cancer cells to utilize one or more of the invasion modes, and to switch among them in response to changing circumstances is termed invasion plasticity and represents a large complication on the road to treating metastatic disease.

Due to the requirements for dynamic changes of cell morphology during invasion, it is evident the invading cell must readily reorganize its cytoskeleton (1–4). This is even more prominent in cells with high invasion plasticity that switch among the elongated mesenchymal and round amoeboid phenotype, in a process termed mesenchymal-amoeboid transition (MAT) or amoeboid-mesenchymal transition (AMT) (5–7). During MAT, cells retract protrusions, round up and initiate intense membrane blebbing, which may be due to loss in cell adhesivity (8) and/or fast increase of hydrostatic pressure that detaches the membrane from the cortex (9). The rapid membrane blebbing is often reduced after transitioning to a motile amoeboid phenotype. Opposingly, AMT is accompanied by loss of blebbing activity and cell elongation through stabilization of protrusions.

Actin reorganization in migrating cells is well described, with RhoGTPases playing a key role (10). Rac and Cdc42 are known to be responsible for promoting actin polymerization leading to the formation of lamellipodia and filopodia as a result of stimulating the Arp2/3 complex through activation of either WASP or SCARE/WAVE family (11–13). Due to its function as an initiator of lamellipodia formation, Rac is preferentially active at the leading edge of migrating cells (14). Opposingly, RhoA activity is higher at the cell rear, where its signaling mediates rear contractility and detachment (15). This is achieved by RhoA-mediated activation of ROCK, which in turn leads to phosphorylation (therefore inhibition) of MLCP, resulting in higher phosphorylation of the myosin light chain and increased contractility (16, 17). ROCK is also responsible for the phosphorylation of LIMK and subsequently of cofilin, which stabilizes actin bundles (18), resulting in formation of stress fibers. Due to the different requirement of protrusive activity and contractility of mesenchymal and amoeboid migration, each is dominated by different RhoGTPase signaling. Amoeboid cells require RhoA/ROCK signaling for their migration and inhibition of this pathway leads to a switch to mesenchymal invasion (19, 20). Similarly, inducing constitutively active RhoA/ROCK can induce the mesenchymal-to-amoeboid switch (21, 22). On the other hand, Rac signaling promotes mesenchymal traits (23, 24).

**Figure 1:**
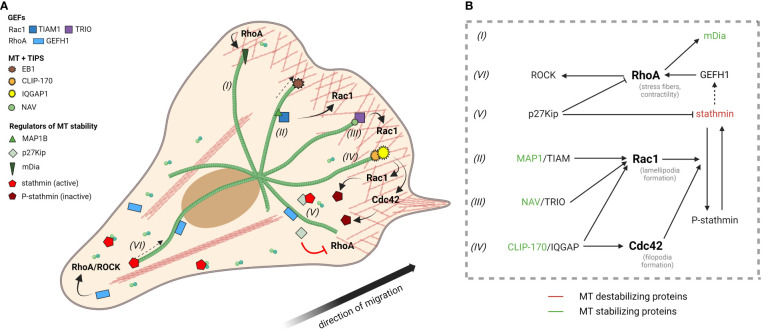
Microtubules and RhoGTPase signaling. **(A)** The length and stability of MTs in migrating cells is interconnected with RhoGTPase signaling and depends on cell polarization. MTs preferentially elongate toward the leading edge, where their growth is enhanced by various MT end-binding proteins and MT stabilizing proteins, which further interact with numerous Rac1 and Cdc42 activators (see TRIO, TIAM and complex IQGAP/CLIP-170). At the leading edge, Rac1 and Cdc42 signaling contributes to actin polymerization (pink network), but also MT stabilization by phosphorylation of stathmin. MT stability at the leading edge is supported by p27^Kip^, which binds stathmin, preventing its activation. Similarly, p27^Kip^ prevents RhoA pathway activation. On the contrary, at the trailing edge, MTs are depolymerized and Rho/ROCK signaling pathway dominates. Stathmin is not phosphorylated by Rac1 or Cdc42 and remains active – able to sequester tubulin dimers and destabilize MTs. MT disruption leads to release, and thus activation, of GEFH1 from MTs into the cytoplasm, where it promotes activation of the RhoA/ROCK pathway. Roman numerals labeling MTs refer to part **(B)** of the Figure. **(B)** Schematic illustration showing interaction between MT stabilizing (green)/destabilizing (red) factors and RhoGTPases. *Created with BioRender.com.*

Moreover, signaling mediated by RhoGTPases interconnects the actin cytoskeleton with the microtubule (MT) network (**Figure 1**). For example, in fibroblasts, MTs growth stimulates Rac1 activity, therefore promoting lamellipodia formation (25, 26). On the other hand, Rac1/Cdc42 signaling can lead to MTs polymerization *via* stathmin inhibition (27, 28), and RhoA can promote stabilization of MTs by its effector, mDia1, which interacts with MTs and induces their capping and alignment with actin bundles (29, 30).

Apart from actin, MTs also interact with intermediate filaments (IFs). This can be either indirectly through linker proteins, such as APC, or directly, and the mutual interaction stabilizes MTs and promotes directed migration (31, 32).

**Figure 2:**
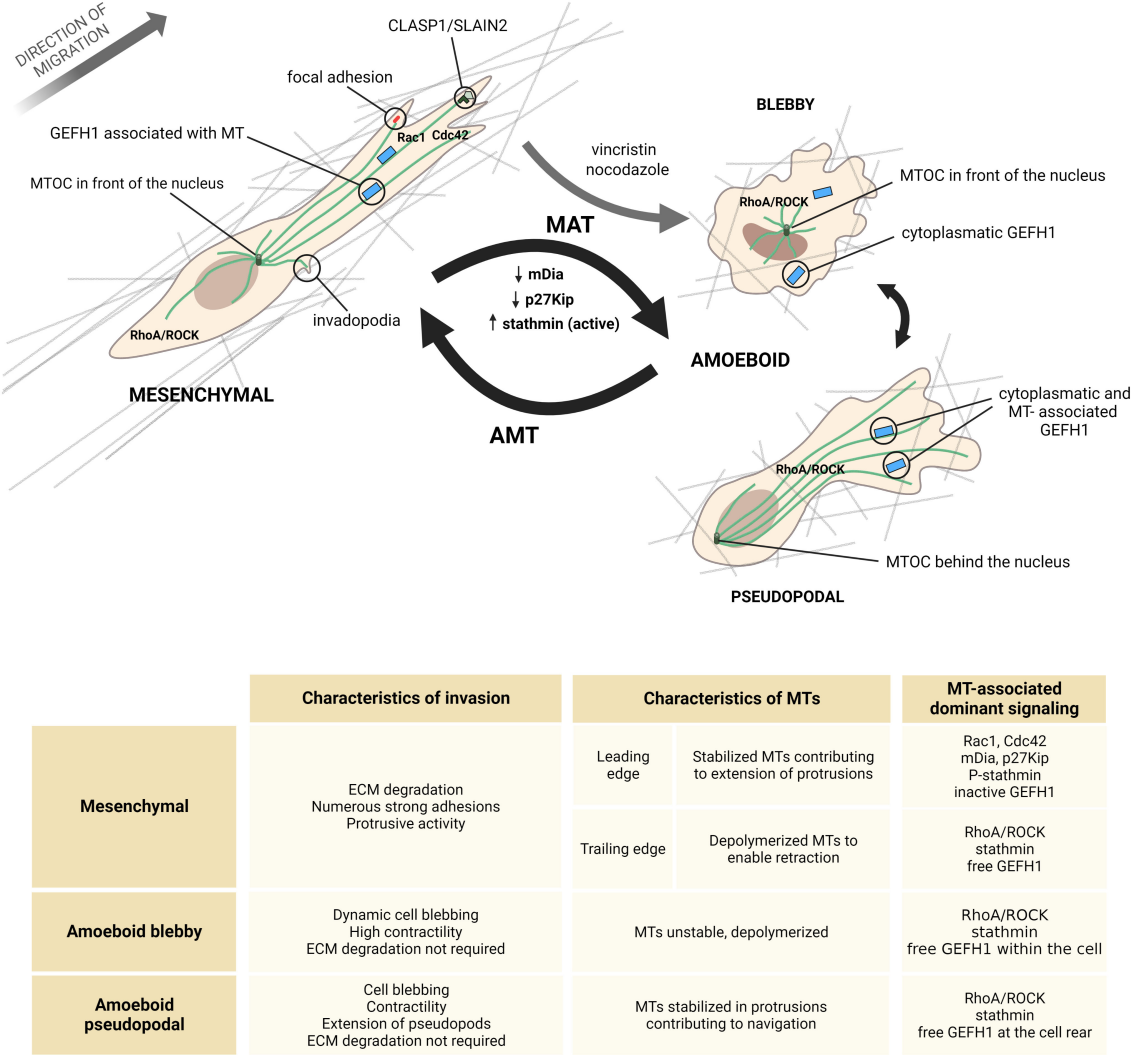
Microtubules in different invasive modes. In mesenchymal cells (left), MTs are elongated and stabilized at the front and depolymerized at the retracting end. Mesenchymal cells contain many adhesion structures, such as focal adhesions and invadopodia, that are tightly coupled to MT dynamics. At the leading edge, protrusive activity is regulated by CLASP and SLAIN, which reduce MT catastrophes and contribute to MT growth persistence and elongation of protrusions. In mesenchymal cells, GEFH1 is predominantly bound to MTs, which keeps it in an inactive state. On the other hand, amoeboid migration modes (right) are generally associated with a less stable MT network. Accordingly, increased stathmin activity or downregulation of MT-stabilizing proteins such as mDia2 or p27Kip has been shown to cause the mesenchymal-amoeboid transition (MAT). In pseudopodal amoeboid cells, MTs contribute to circumnavigation and pseudopod extension. In blebby amoeboid cells, MTs are generally disrupted and thus MT-destabilizing drugs, such as vincristine and nocodazole, promote transition to the blebby amoeboid phenotype. In amoeboid cells, GEFH1 can be found free in the cytoplasm, where it activates the RhoA/ROCK pathway. Note that the MTOC is located in front of the nucleus in both mesenchymal and amoeboid cells, although in some pseudopodal amoeboid cells, such as leukocytes, the MTOC is found at the cell rear. This information, together with the basic characteristics of the individual invasion modes, is summarized in the table at the bottom of the figure. *Created with BioRender.com.*

The role of microtubules in cell migration is multifaceted, encompassing intracellular transport and delivery of migration associated cargo, protrusion stabilization and regulation of adhesions (33–35). Less is known about the role of microtubules in 3D migration, yet alone specifically in amoeboid or mesenchymal invasion. Thus, we would like to summarize current knowledge on the role of microtubule cytoskeleton in cell invasion in the context of mesenchymal and amoeboid phenotypes and transitions among them (**Figure 2**).

## 2 Microtubule associated proteins in cell migration

The stability and dynamics of the MT network are modulated by numerous MT associated proteins, some of which have been directly linked to cell invasion plasticity, see below and in **Figure 1**.

### 2.1 EB1

End binding protein 1 (EB1) binds +end of MTs, localizing to the distal tips of MTs, the centrosome or MT ends in the mitotic spindle. It is sometimes referred to as the „master regulator of plus-end-tracking proteins (+TIPs)” for its ability to recruit various +TIPs and thus influence not only MTs themselves, but other processes such as membrane-anchoring or actin polymerization as well (36). EB1 binding to MTs stimulates MT elongation, whereas its dissociation causes slower growth of MTs and can influence cells’ direction of movement (37). Interestingly, depletion of EB1 has much bigger consequences on migration and protrusion branching in 3D migration. Whereas in 2D there is no significant effect on protrusions and overall migration speed, in 3D, depleting EB1 in mesenchymally migrating cells results in slower invasion and defects in cell directionality (38). In mesenchymal cells, EB1 mediates the binding of proteins CLASP1 and SLAIN2 to MTs, which contributes to their growth persistence in protrusions by reducing catastrophes (**Figure 2**). This was shown to be necessary for the invasive shape and 3D mesenchymal invasion (39).

### 2.2 IQGAP

IQ motif-containing GTPase-activating protein 1/2/3 (IQGAP1-3) are scaffold proteins that integrate many signaling pathways *via* direct and indirect binding of over 90 proteins (40), including RhoGTPases (**Figure 1**).

IQGAP proteins directly affects the dynamics of both the actin and the microtubule cytoskeleton. IQGAP1 is able to cross-link F-actin filaments (41–44) or by binding barbed ends of actin filaments, inhibit their growth, and protect them from depolymerization (42). IQGAP1 interacts with N-WASP and Arp2/3 forming a complex able to nucleate branched actin filaments (45, 46). In agreement, IQGAP1 was shown to be localized at the leading edges of polarized cells and in the lamellipodia of motile cells (43, 44, 46–48). One of the main functions of IQGAP1 is anchoring MTs to the cell cortex enabling directional movement.

With regard to MTs in migration, one of the main functions of IQGAP1 is anchoring MTs to the cell cortex enabling directional movement, a process regulated by multiple mechanisms. IQGAP1 interacts with +TIP CLIP-170 and activated Rac1 and Cdc42 (but not RhoA) forming a tripartite complex leading MTs to the cortex to areas with activated Rac1/Cdc42. Impaired binding of IQGAP1 caused by mutation at its C-terminus results in multiple leading edges in cells (49, 50). In addition, IQGAP1 and active Rac1/Cdc42 form a complex with adenomatous polyposis coli (APC) cortical filaments and depletion of either APC or IQGAP1 inhibits polarized migration (51). Another +TIP linking IQGAP1 to MTs at the cell cortex is protein SKAP (+TIP known to bind to EB1), yet again, disrupting the interaction of SKAP and IQGAP1 impairs cell migration (52).

Overall, IQGAP1 overexpression can promote cell migration and neurite outgrowth, while its depletion leads to decreased cell migration (47, 53, 54), providing evidence that the actin/microtubule crosstalk is necessary for polarized, directed cell movement.

### 2.3 Navigators

Navigator proteins 1-3 (NAV1/2/3) are microtubule + end binding proteins that are implicated in axon guidance and neurite outgrowth in the brain (55, 56). In neurons, NAV1 is localized at the neurite tips where it binds actin-rich domains and crosslinks with the MTs in an EB1-dependant manner (55, 57). It also forms a complex with the protein TRIO, a guanine nucleotide exchange factor (GEF) known to activate Rac1 and RhoG (56–59). Thus, NAV1 does not influence MTs polymerization per se, but it promotes +end rescue and prevents catastrophes in the F-rich-domain periphery. Cells depleted in NAV1 were shown to be defective in migration during embryonic development (55). Similarly, NAV3 binds to MTs +end *via* EB1 and increases their polarized growth in response to EGF signaling in cancer cells by protecting them from catastrophes. Cells overexpressing NAV3 displayed higher MTs acetylation and resistance to nocodazole treatment, both distinctive of stabilized MTs (60). On the other hand, cells depleted in NAV3 showed more random migration and lost the ability to migrate persistently after EGF induction (60). Since persistent, protrusion dependent migration is characteristic of mesenchymal cells, Navigators are likely to be more crucial for mesenchymal than amoeboid migration.

### 2.4 Stathmin 1

Stathmin, also known as oncoprotein 18 (Opt18), is an important microtubule destabilizing protein able to cause MT depolymerization. Two modes of action have been described – first, by stimulating MT catastrophes (61), and second, by sequestrating αβ tubulin dimers to prevent their assembly (62) - which seems to depend on environmental conditions (63).

Stathmin is regulated by several kinases, and its phosphorylation on at least one of its four serins suppresses its destabilizing activity (64, 65). Moreover, Stat3 can bind to stathmin at its tubulin-binding site to prevent its function (66). Similarly, p27Kip (see further) binding prevents stathmin-regulated MT destabilization (67, 68). Of note, p27Kip expression is partially regulated by Stat3 (69).

The regulation of stathmin phosphorylation is key for cell migration, as it enables creation of a gradient of MT stability from the leading edge to the trailing edge. At the leading edge, high levels of Cdc42 and Rac1 prevent stathmin activation (27) resulting in stabilized MTs in these parts of the cell. On the trailing edge stathmin is not phosphorylated by Cdc42 or Rac and its MTs-destabilizing activity increases, leading to MT network disassembly and proper contraction of this end during cell movement (70) (**Figure 1**).

Generally, stathmin is a strong pro-migratory factor as evidenced by a number of studies (67, 71), although in in certain conditions its inhibition may stimulate migration (66). This may be dependent on whether the cells utilize the mesenchymal and amoeboid type of invasion, since stathmin SQ18E, which is unable to undergo inhibitory phosphorylation, was directly linked to promotion of the round, amoeboid-like phenotype in sarcoma cells (71) (**Figure 2**).

### 2.5 P27^Kip^

Protein p27^Kip^ is mainly known for its nuclear role as a cyclin dependent kinase inhibitor and thus inhibitor of cell cycle progression (72). However, it also plays an important role in the cytoplasm where it interacts with other proteins through its C-terminal domain to modulate cell motility and tumor progression (67, 73, 74). One of the interaction partners of p27^Kip^ is stathmin, binding of which inhibits stathmin´s activity leading to more stable MTs. In mesenchymal cells, this interaction limits migratory potential (67, 68) (**Figure 1**).

P27^Kip^ does not influence only the MT network, it also affects actin filament reorganization. It was shown that p27^Kip^ is able to interact with RhoA, inhibiting its ability to bind GEFs and thus preventing its activation (73). Cytoplasmatic p27^Kip^ affects the actin cytoskeleton also indirectly *via* Rac1 dependent actin rearrangement and polymerization, leading to an increase in cell migration (75).

Of note, transformed fibroblasts lacking p27 ^Kip^ adopted a rounded morphology with cortical actin formation and loss of β1 integrin clusters, corresponding to the mesenchymal-amoeboid transition (76). Here both the actin and MT cytoskeleton were altered, showing a synergic effect of p27 ^Kip^ in regulation of cell invasion. In macrophages, p27 ^Kip^ contributes to the onset of mesenchymal migration by inhibition of RhoA/ROCK and lack of p27 ^Kip^ promotes amoeboid migration (77). Collectively, cytoplasmic p27 regulates invasion plasticity and exerts pro-mesenchymal signaling (**Figure 2**).

### 2.6 mDia2

mDia2 protein (mammalian homolog of Drosophila diaphanous; also known as DIAPH3) belongs to the group of formins (78), which are proteins involved in actin nucleation and elongation. In addition, mDia2 is able to bind and stabilize MT polymers. Its silencing leads to MT catastrophes and rewires EGFR and ERK signaling, resulting in increased ameboid traits (79). A different study showed that mDia2 in complex with its inhibitor induces amoeboid morphology in cells (80). Accordingly, depletion of mDia2 promoted individual dissemination of amoeboid cells from tumor spheres (81). Importantly, this affects cancer treatment, since cells without mDia2, exerting unstable MTs and amoeboid characteristics, are more susceptible to taxane chemotherapy (82). Overall, mDia2 regulates invasion plasticity through MT stabilization and actin nucleation, and its absence promotes the amoeboid invasion phenotype (**Figure 2**), suggesting its pro-mesenchymal role.

### 2.7 GEFH1

An important molecule which interconnects MT dynamics and the actomyosin network is guanosine exchange factor H1 for GTPase RhoA (GEFH1), also known as ARHGEF2. GEFH1 is a one of the few GEFs that associate with polymerized microtubules. Upon MT depolymerization, GEFH1 is released and its guanosine exchange activity increases, leading to higher activation of RhoA and its downstream signaling (**Figure 1**). This mechanism regulates RhoA activity in different parts of the migrating cell based on MT dynamics (83). Of note, GEFH1 activity can be further potentiated by phosphorylation mediated by Src kinase at the protruding cell edge (84). GEFH1 also affects focal adhesions (FAs) turnover in migrating cells and dysfunctional GEFH1-RhoA activation can disrupt cell motility (83). In lymphoma cells, Stat3 signaling induced destabilization of MTs, which led to the release of GEFH1 and as a result, amoeboid invasion (85).

In summary, the GEFH1-RhoA signaling pathway is induced by MTs destabilization, and its extent promotes either mesenchymal or amoeboid migration. In mesenchymal cells, activation of this pathway by dynamic growth of MTs contributes to protrusion regulation and faster FA turnover. In cells with disrupted MTs, GEFH1-RhoA signaling dominates and induces amoeboid invasion through the RhoA/ROCK pathway (**Figure 2**).

### 2.8 MAPs (MAP1B, MAP4, MAP2)

There is also a large group of microtubule associated proteins (MAPs) that bind to MTs, but surprisingly, despite their unified function of stabilizing MT, they have different roles in tumor progression and may function as both pro- and anti-metastatic factors (86–88). We hypothesize that this inconsistency may be due to the different dependency of the amoeboid and mesenchymal migration on the MT network. Specifically, a change in invasive behavior was connected to MAP1B, MAP2, MAP4 and MAP7. Although there is no direct link between invasion plasticity and MAPs described so far, they likely participate by indirect signaling affecting RhoGTPases signaling. For example, MAP1B increases Rac1 activity through interaction with TIAM-1, a Rac1-GEF (89). Another possible mechanism involves phosphorylation of MAP4, which inhibits its MT stabilizing activity (90) and leads to MT disruption, which can increase RhoA through GEHH1 release (91).

### 2.9 Microtubule organizing center in invasion

For efficient cell migration, cells need to be polarized to maintain directionality of movement. The polarity of the cell is determined, amongst others, by the position of the nucleus and centrosome, forming the nuclear-centrosomal axis. By moving the centrosome, also known as the microtubule organizing center (MTOC), to a different position within the cell, the polarity of a cell can be shifted (92).

When cells gain a mesenchymal migratory phenotype, the centrosome position shifts to a central position in front of the nucleus relative to the future movement of the cell (93). However, this positioning of the centrosome is not a rule, as some studies show localization of the centrosome behind the nucleus (94, 95). Some studies also show that the position of the centrosome is influenced by the geometrical limitation of the cell’s surroundings rather than its function (96).

In most amoeboid cells, the location of the MTOC corresponds to mesenchymal cells. Interestingly though, in amoeboid leukocytes, the MTOC is placed behind the nucleus toward the cell rear (**Figure 2**) (97, 98) and participates in the path finding mechanism. Once the cell’s nucleus, which represents the bulkiest part of the cell, and the associated MTOC successfully pass through a pore, protrusions directed to the smaller pores are retracted and the cell continues its movement through the path of least resistance. In this case, disrupting MTs leads to loss of cell coherency and results in fragmentation of the cell (97).

## 3 Microtubules and cell adhesive structures in invasion

One of the main distinctions between ameboid and mesenchymal migration is their adhesion-dependency. Unlike ameboid cells that do not require stable ECM attachment for their movement, mesenchymally migrating cells establish numerous interactions with the ECM, including the formation of integrin-based adhesions and proteolytically active structures that enable contact-driven invasion (**Figure 2**) (99–101). Microtubules play a role in regulation and dynamics of these adhesive structures and are therefore an integral part of signaling regulating adhesion-dependent invasion.

### 3.1 Focal adhesions

FAs are integrin-based structures responsible for strong adhesion of cells to the ECM. Importantly, they also transmit information about the surrounding environment such as its stiffness by signaling to cytoskeleton associated proteins (102–104). Both Rac and RhoA play role in FA regulation – whereas Rac activity is prominent in the formation of FAs, RhoA activity and Rac inhibition are needed for the maturation of FAs (105, 106).

The main role of MTs within FAs is the transport of integrins and metalloproteases (MMPs) to the cell membrane and the regulation of FAs turnover (107). Although there is no evidence of direct binding of MTs to FAs so far, MTs are known to be guided towards them and anchored in their proximity (108–111). The targeting of mature FAs by MTs results in FA disassembly and cell edge retraction, and preventing the contact between MTs and FAs leads to enlarged FAs (112). In agreement, nocodazole-induced MTs depolymerization also results in larger FAs, whereas MT regrowth after nocodazole washout disassembles FAs (112–114). Nevertheless, these studies were done in 2D environments, where the structure, composition and dynamics of FAs is different than in 3D environments (115), and many mechanisms valid in 2D systems are yet to be verified for 3D migration.

### 3.2 Podosomes

Podosomes and invadopodia are similar actin-based structures that play role in cell migration and ECM degradation, which is a key feature of mesenchymal invasion. Whereas podosomes are small, dot-like dynamic structures at the leading edge or organized rings exhibiting shallow ECM degradation, invadopodia are larger, irregularly shaped clusters usually localized in the central area of the cell. They are less dynamic and form outstretched extensions into the matrix resulting in deeper and more focused ECM degradation (116).

An intact MT network is necessary for the formation of podosomes since MT depolymerization (induced e.g. by nocodazole) leads to podosomal disassembly (117, 118). MTs are directed to podosomes through +TIPs such as EB1 and CLASPs (119, 120). Targeting of podosomes by MTs is associated with their higher dynamics, and podosomes without MTs show increased stability similarly to FAs (109, 112, 121).

The lifespan of MTs in podosomal structures is regulated by RhoGTPases. Inhibition of RhoA is able to increase the stability of MTs in podosomes, promoting podosome belt assembly (122). Accordingly, activation of RhoA leads to podosome disassembly through the RhoA/ROCK/MLCP axis as actomyosin contractility increases podosomes turnover and their disassembly (123–125). Altering Cdc42 and Rac1 activity was also shown to disrupt podosomes (118, 122, 126).

### 3.3 Invadopodia

Invadopodia are invasive structures commonly found in various cancers (127). Unlike podosomes, microtubules are not required for invadopodia formation but are necessary for their elongation and correct function (128, 129). Invadopodia first form as a smaller actin-based structure with microtubules excluded from their core (130). Only after maturation of invadopodia, intermediate filaments and microtubules (typically 1-2 MTs per protrusion) invade their structure and allow invadopodia elongation. The microtubules that invade the shaft of invadopodia are stable, whereas at the base of the invadopodium more dynamic MTs are found (128). Accordingly, MT depolymerization does not affect the formation of small invadopodia, but limits their elongation and maturation (129).

One important role of MTs in invadopodia is the polarized trafficking of components such as matrix MMPs to the cell membrane (128, 131, 132). Moreover, the MT associated protein IQGAP1 accumulates in invadopodia where it interacts with exocyst components, and this interaction promotes invadopodia proteolysis by accumulation of MT1-MMP in a MT-regulated manner (133). Also, the exocytosis of MMP2 and MMP9 in melanoma cells is MTs-dependent (132) supporting the role of MTs in trafficking invadopodia components.

## 4 Microtubules and ECM conditions

It is well known that the conditions of the surrounding environment and its physical properties largely influence the choice of the migration mode. Amoeboid cells favor the large pores found in less dense ECM (134, 135) or confining conditions (100, 136), while mesenchymal cells with their proteolytic activity are able to migrate through stiff ECM and take advantage of the increased number of adhesion sites (101, 135, 137). Unsurprisingly, the MT network is receptive to ECM cues *via* posttranslational modifications that respond to ECM stiffness such as acetylation and glutamylation.

Generally, acetylated MTs are more stable and resilient. The acetylation of α tubulin is driven by α tubulin N-acetyltransferase 1 (αTAT1), and opposingly, histone deacetylase 6 (HDAC6) elicits tubulin deacetylation. Confusingly, both acetylation and deacetylation have been linked to accelerated cell migration. αTAT1 stabilizes MTs at the leading edge of migrating cells to promote cell motility and tumor progression (138). HDAC6 elicits pro-invasive signaling by activating Rho family GTPase, specifically by Rac1 (139). In addition to MTs, αTAT1 and HDAC6 are able to acetylate/deacetylate other substrates, such as cortactin. Cortactin contributes to actin filament assembly and is also required for MT1-MMP delivery to the leading edge of migrating cell (140), a process important for mesenchymal migration of tumor cells (141).

A recent study describes that ECM characteristics, MT dynamics and cell metabolism are interlinked to regulate cell invasion. Stiff substrates induce the conversion of glutamine to glutamate, increasing MT stability by glutamylation, resulting in an invasive phenotype and metastasis of breast cancer cells (142). However, a different study showed that higher density of collagen destabilizes MTs and induces GEFH1 mediated RhoA signaling (143).

Another characteristic of the surrounding environment that affects cell migration is oxygen availability. Hypoxia leads to MT depolymerization as a consequence of MAP4 and stathmin phosphorylation (144, 145). Subsequently, MTs depolymerization promotes RhoA activity by releasing GEFH1, a mechanism known to promote amoeboid invasion. In agreement, hypoxic conditions trigger the collective-amoeboid transition (146).

### 4.1 Microtubule targeting drugs

Microtubule drugs, due to their immense effect on MT structure and dynamics, have extensive impact on cell behavior. They are commonly used as chemotherapeutic agents based on their ability to arrest cell proliferation and cause cell death (147). Nevertheless, MT drugs exhibit more extensive behavior than just antimitotic effects, including deregulation of cell migration (148). They also interfere with invasion plasticity manifesting different effects on each invasion mode. Drugs inhibiting MT polymerization promote the amoeboid mode as it is less MT-dependent (**Figure 2**). For example, vincristine which is able to sequester tubulin dimers and prevent MT polymerization, induces the amoeboid phenotype through GEFH1/RhoA signaling (149). Similar results were observed with nocodazole, which also leads to MT disassembly and subsequent RhoA activation (150). In fibroblasts, nocodazole treatment prevented cells to adopt an elongated morphology and instead induced a round morphology, typical of the amoeboid phenotype (151). Treatment of cells with paclitaxel (taxol) stabilizes microtubules, but does not promote mesenchymal migration, instead it induces a non-motile phenotype (152–154). In lymphocytes that utilize the amoeboid migration mode, taxol treatment inhibited migration in both 2D and microstructured environments, whereas nocodazole treatment increased membrane blebbing without increase in migration in 2D (155), but was able to promote amoeboid invasion in 3D (156).

The wide clinical usage of classic MT drugs is hampered by notable drug resistance and toxicity, fueling the chase for novel compounds with improved characteristics (157), many of which are in clinical trials (158). Moreover, in recent years it has become evident that the requirement for anti-metastatic behavior should be evaluated as well as primary growth shrinkage (159–162). In point of fact, the prototypic antimitotic drugs paclitaxel and vincristine can in certain instances promote metastasis (163, 164). On the other hand, vinorelbine treatment in mice reduced metastasis more effectively than primary tumor growth (165). Another microtubule inhibitor, eribulin, exerts migrastatic behavior both in experimental conditions (166, 167) and in patients with advanced metastatic breast cancer (168).

Taken together, a suitable combination of microtubule drugs with invasion specific inhibitors could synergically target cell proliferation and both amoeboid and mesenchymal invasion, i.e., elicit both anti-proliferative and migrastatic effects.

## 5 Concluding remarks

The invasion phenotype is a result of the orchestration of all three cytoskeletal systems. Here, we have summarized evidence that MT dynamics can directly affect cancer invasion plasticity and described its role in both amoeboid and mesenchymal cells (**Figure 2**). Nevertheless, the contribution of the individual cytoskeletal components specifically during the transitions between amoeboid and mesenchymal invasion modes remains to be described. For example, it is not clear whether during MAT the disintegration of MTs precedes, follows, or accompanies formation of the actomyosin cortex. Moreover, the cells´ reaction to the rearrangement of the MTs network is dependent on several factors including differences between 2D and 3D environments, cell type, RhoGTPase signaling or presence of MT drugs, and thus the role of MTs in invasion is not uniform.

Above mentioned evidence shows that amoeboid migration is possible even if MTs are unstable or depleted and destabilization of MTs can directly induce amoeboid invasion. This is contrary to the finding that MTs are retained in amoeboid leukocytes and in fact promote migration by contributing to path-finding mechanisms or protrusion retraction (97, 169). These seemingly contradictory findings may be explained by the existence of multiple types of amoeboid invasion that include blebby, stable-bleb and pseudopodal subtypes that differ in their extent of adhesion, and protrusive and contractile activity, which occur based on cell type and/or ECM conditions (99, 170, 171). For example, leukocytes are known to adopt the pseudopodal amoeboid mode, which is dependent on MT and actin-driven pseudopod extension. On the other hand, the highly contractile bleb-based amoeboid modes are reliant on actomyosin activity, but do not require MTs (171) (**Figure 2**). Interestingly, the varying structure of the MT network in amoeboid cells is reflected also in Amoebozoa, unicellular protists after which the amoeboid migration mode is named. Based on immunocytochemistry staining of MTs, in some amoebae MTs are present as short cytoplasmic fibers, other contain long, parallel MT bundles and in some cases fibrous MTs where not detected at all (172). It thus seems that indeed forms of amoeboid migration dependent and independent on MT network exist.

On the other hand, mesenchymal migration requires the role of MT for multiple processes. Especially at the leading edge MTs contribute to extension and stabilization of protrusions, but also to formation of adhesive and proteolytic structures. However, pharmacological stabilization of MTs limits migration and invasion of cells, suggesting that excessive stabilization halts migration altogether.

In this context, it is not easy to conclude whether increased depolymerization of MTs leads to higher invasiveness or the opposite. On the contrary, what we can confirm is that MT dynamics directly affects the ability of the cell to choose among the invasive modes that are most profitable for them under the given conditions.

## Author contributions

AŠ, DR, and JB contributed to the conception and processing of the article. AL, AŠ, and MP wrote the original manuscript. AL and AŠ contributed significantly to the first design of the images, AL created the images and all authors contributed to their finalization. All authors contributed to the article revision and approved the submitted version.
